# The development of the social health scale for the elderly

**DOI:** 10.1186/s12955-018-0899-6

**Published:** 2018-04-18

**Authors:** Chengzhen Bao, Zhebin Yu, Xuefen Yin, Zhen Chen, Lu Meng, Weibo Yang, Xueyu Chen, Mingjuan Jin, Jianbing Wang, Mengling Tang, Kun Chen

**Affiliations:** 1 Department of Epidemiology and Health Statistics, Zhejiang University School of Medicine, Zhejiang, Hangzhou China; 2 Community Health Service Center of Mishi Lane, Gongshu District, Zhejiang, Hangzhou China; 3 Health and Family Planning Commission of Xihu District, Zhejiang, Hangzhou China; 4 Zhejiang University School of Medicine, Zhejiang, China

**Keywords:** Social health, Elderly, Scale, Norms

## Abstract

**Background:**

With the elderly population comprising an increasing large proportion of society, a valid and reliable measure of social health in the elderly is indispensable for a comprehensive health assessment. The objective of this study is to develop a Social Health Scale for the Elderly (SHSE).

**Methods:**

A draft scale was generated based on a literature review and expert surveys. Pilot testing was conducted from December 14, 2015, to January 8, 2016. Some items were removed after assessment with five statistical analysis methods. Field testing began on November 6, 2016, and ended on January 20, 2017. After field testing, the reliability and validity of the scale were assessed and the norms in Hangzhou were calculated.

**Results:**

In the two tests, 430 and 2404 subjects were included in the statistical analyses. The long form of the SHSE (SHSE-L) contained 25 items, 14 of which were also in the short form (SHSE-S). The internal consistency of the SHSE-L was acceptable. The test-rest reliability and inter-rater reliability were moderate, but the concurrent validity, construct validity, and convergent and discriminant validity were desirable in both versions. The standard and percentile rank norms in Hangzhou, China were developed based on the field testing data.

**Conclusions:**

The population-based social health of the Chinese elderly can be validly and reliably assessed with the SHSE.

**Electronic supplementary material:**

The online version of this article (10.1186/s12955-018-0899-6) contains supplementary material, which is available to authorized users.

## Background

Population ageing is quickly becoming a problem worldwide. The World Health Organization (WHO) reported that there are currently 900 million people aged 60 years and older, which may increase to 2 billion by 2050 [1]. Furthermore, in 2050, approximately 80% of the elderly are predicted to live in countries that are currently low- or middle-income [2]. The World Health Statistics reported that the life expectancy in most countries was greater than 60 in 2015, and the global average life expectancy was 71.40 years [3]. The mortality rate of the elderly is decreasing, which is the primary reason for the increasing life expectancies in high-income countries [4]. Although there have been considerable research developments regarding the medical and public health of the elderly, the health status of the elderly is not significantly better than that of their parents [1].

However, the definition of health is no longer merely the absence of diseases. The ability for routine functioning is also important and should be given proper attention when assessing health status [1]. The WHO has stated that “health is a state of complete physical, mental and social well-being and not merely the absence of disease or infirmity” [5]; however, the requirement of “complete…well-being” does not apply to the aged population. Many elderly individuals with one or two chronic diseases consider themselves “well enough” to be aging successfully, which refers to a status characterized by a low probability of diseases and related disabilities, high cognitive and physical functioning, and active social engagement [6, 7]. Therefore, a specialized measurement of health status for the aged population should be developed separately for an accurate description of elderly health status.

It is more difficult to establish the norm of social health compared to that of psychological or physical health [8]. Social health contains two aspects: individual social health and the social health of society or a population [9]. Social health of an individual is usually explained as “well-being”, “adjustment” or other terms rather than “health” [10], and it can be measured from two aspects: social support (SS) and social adjustment (SA). The assessment of SS mainly discusses the processes and outcomes of support from relatives, friends or other people. The measurement of SA usually refers to relationships with others and the performances of social roles [9]. SS places emphasis on the level of social support the subject receives from others whereas SA focuses on the adaptive capacity of subjects to actively interact with the community where they live. Some studies have assessed the relationships between SS, SA and other health outcomes and reported that SS was a significant factor contributing to loneliness in the elderly [11]; moreover, emotional support has a positive effect on reducing the mortality of the elderly [12]. Some researchers have mentioned that SA is related to quality of life [13] and that psychotherapy is effective for improving the SA of elderly individuals with suicide attempts [14].

Another important tradition of social health assessment is the characteristics of society, that is, the social health of the society as a whole. A healthy society is defined as follows: “A society is healthy when there is equal opportunity for all and access by all to the goods and services essential to full functioning as a citizen” [10]. In addition, previous studies have indicated that the neighbourhood environment can significantly influence the psychological and physical health of the elderly [15]. Therefore, the “social health of society” mainly reflects the neighbourhood environment. The utilization of health services was partly determined by the perceived health status [16]. Similarly, the utilization and feeling of the same objective environment might be different between any two people [17] and is influenced by their demands and criterions. Instead of objective environmental indicators, perceived environmental indicators are more suitable for assessing the support received from the environment. Therefore, to assess the social health of society, this study took perceived environment resources (PERs) into account, which refers to perceived built environment, community management and service. The relationships between PER and health outcomes have been reported, and previous studies have demonstrated that PER was marginally associated with greater possibilities of poor self-rated health [18] and was associated with depressive symptoms, anxiety and physical symptoms [19].

To improve the health status of the Chinese elderly, the development of a specialized and comprehensive measuring tool that can accurately evaluate the social health status of the Chinese elderly is required. Social health is an important part of health. However, a measuring tool for the Chinese elderly has not been previously developed. This study aimed to develop a scale to assess the social health status of the elderly that evaluated both the social health of the individual (SS and SA) and the social health of society (PER). The scale could contribute to a more comprehensive measurement of the health status of Chinese elderly.

## Methods

### Design

We developed the Social Health Scale for the Elderly (SHSE) over 4 phases, which are discussed in detail below.

#### Phase 1

Based on the literature review findings, the items in the original draft scale were chosen. Some items were excluded after consulting with experts, and a revised version of the draft scale was developed.

#### Phase 2

Pilot testing aimed at selecting the items for the revised draft scale. In this phase, a test-retest reliability analysis, Cronbach’s alpha analysis, a correlation analysis, a distinguishability analysis and a principal component analysis were conducted for item selection, and then the final versions (some items in the long form were deleted in the short form) of the SHSE were generated.

#### Phase 3

Field testing was conducted to assess the validity and reliability of the scales (SHSE-L: long form of the SHSE; SHSE-S: short form of the SHSE). The test-retest reliability, internal consistency reliability, inter-rater reliability, concurrent validity, construct validity, convergent validity and discriminant validity were calculated in this phase.

#### Phase 4

Based on the field testing data, the raw score distributions among the different groups could be compared, and two norms (standard norm and percentile rank norm) of social health were generated.

### Development of the draft scale

The draft scale was generated by reviewing published books, systematic reviews and original articles [9, 15, 20–22]. Objective evaluation indicators, such as the frequency of communication with children and duration of optimistic mindset, were considered the better choices. The item pool included items related to social health as much as possible, and each question intended to reflect a specific aspect of some items.

After consulting with sociology experts and public health experts, the items in the original draft scale that contained repeated content or were not suitable for the Chinese elderly were deleted, and necessary missing items were added. The questions and options were modified for better intelligibility.

### Data collection

Before the pilot testing, a trial survey was conducted to test the investigation ability of the interviewers after training. Each interviewer was required to participate in standardized training and then normatively interviewed at least one person who was aged 60 years or older. Four communities in the Gongshu district were randomly selected. The Gongshu district is located in the centre of Hangzhou, and the proportion of elderly individuals there is similar to that in Hangzhou as a whole [23]. The minimum sample size was calculated to ensure that there were at least 10 subjects per item in the factor analyses [24]. The target population was the general healthy population aged 60 years and older. After the Health Records in community public health service stations were checked, persons who were bed-ridden, had serious physiological or psychological illnesses, and/or had hearing disorders, were excluded before sampling. Then, stratified random sampling by age and gender was conducted. The community doctors contacted potential participants by telephone before conducting the interviews to obtain higher resident compliance. Each participant was required to sign informed consent if he or she agreed to be an interviewee. The interview was conducted at the Community Health Service Centre of the community that the participants lived in, and the participants were required to attend the interview in person to complete a face-to-face interview. During the interview, if the interviewer believed that the characteristics of this participant met the exclusion criteria, the data of this interviewee were not included. Those participants who did not attend the interview in time but did not refuse to participate were contacted by telephone more than once because the elderly might forget the designated interview appointment time because of their poor memory.

The field testing procedure was similar to that of the pilot testing. The main differences were the field and the method of sampling. Considering the compliance and the number of aged residents, eight communities in Gongshu district and nine villages in Xihu district were selected. The former was the sample source of urban residents, and the later was that of rural residents. The sample size of each district should be 40 times larger than the number of items in the final version of the SHSE-L [25]. Convenience sampling was used for field testing. Convenience sampling refers to a procedure in which community doctors contact potential participants in advance of the interview, followed by the interviewers remaining in the field for one week or less to interview participants. Those residents who did not participate in the interview in time but did not refuse to participate were reminded by telephone calls, but the interviewers would not wait for them if they did not come to the site for the interview within the stipulated time. The chi-square test was used to compare the distributions of the subjects in the two tests.

### Item selection

After calculating the raw scores of the revised draft scale, we selected items to generate the final versions of the SHSE (SHSE-L and SHSE-S). We utilized five statistical methods to select the items in the revised draft scale.

#### Test-retest reliability analysis

The interval between the test and re-test did not exceed two weeks [26, 27]. The correlation coefficient between the raw score of a particular item in the first interview and that in the second should be larger than 0.30 (*P* < 0.05) for this item to be retained. If the correlation of some item was too small or the *P-value* was not less than 0.05, then the test-retest reliability of this item was unsatisfactory.

#### Cronbach’s alpha analysis

We calculated the standardized Cronbach’s α coefficients of this scale before and after eliminating some items. If the standardized Cronbach’s α coefficient of the scale increased after eliminating some items, then these items were deleted to obtain better internal consistency of the scale [28].

#### Correlation analysis

The raw score of some items should statistically relate to that of the related dimension (*r* > 0.40, *P* < 0.05). Meanwhile, each remaining item should be statistically unrelated (*P* ≥ 0.05) or minimally related (*r* < 0.30) to the other two unrelated dimensions.

#### Distinguishability analysis

We compared the raw scores of a particular item between the high-score group (P_75_) and the low-score group (P_25_). An item was determined to lack distinguishability when the difference in distribution was not statistically significant (*P* ≥ 0.05).

#### Principal component analysis

A principal component analysis was used to extract the factors after performing Bartlett’s test and using the Kaiser-Meyer-Olkin (KMO) measure (Bartlett’s test: *P* < 0.05; KMO > 0.60) [29]. The number of factors was preset and was equal to the number of sub-dimensions (see Table 1) because we considered that the sub-dimensions were reasonable and could independently explain the social health of the Chinese elderly. The factors were rotated by Varimax because each two items (see Table 1) were not significantly correlated (the correlation coefficient of each two items was less than 0.30, or *P* ≥ 0.05). Items were reserved if the factor loadings were greater than or equal to 0.40 [30].

**Table 1:** The draft structure of Social Health Scale for the Elderly

Dimensions	Sub-dimensions	Items (variables)	Ref.
Social support	Emotional support	Being listened to (B01)	[1–3]
		Being accepted totally (B02)	[1]
		Being understood (B03)	[3]
		Being supported in major decision (B04)	[1]
		Being emotional cared for (B05)	[1, 4]
		Being accompanied (B06)	[3]
		Being comforted (B07)	[1]
		Being moved (B08)	[3, 4]
	Informational support	Being given useful information (B09)	[3]
		Being reminded to improve (B10)	[1]
		Being given useful suggestion (B11)	[1, 3, 4]
	Instrumental support	Being helped with daily chores during illness (B12)	[3, 4]
		Being given financial aid (B13)	[4]
		Being given material aid (B14)	[4]
		Being cared in daily life (B15)	[3]
Social adjustment	Social participation	Working for pay (B16)	[5]
		Doing housework (B17)	[5]
		Doing volunteer work (B18)	[5]
		Participating in collective recreational activity (B19)	[5]
Social adjustment	Social relationship	Communication with children (B20)	[5]
		Communication with friends (B21)	[5]
		Communication with other relatives (B22)	[5]
		Relationship with neighbors (B23)	[5]
		Relationship with partner (B24)	[5]
		Abnormal psychology in social contact (B25)	[5, 6]
		Feeling about others (B26)	[5]
	Ego system	Self-health concern (B27)	[5, 6]
		Interests and hobbies (B28)	[5]
		Family financial secured (B29)	[5]
		Attitude towards life (B30)	[6]
Perceived environment resource	Natural environment	Quality of natural environment (B31)	
	Built environment	Manufactured landscape (B32)	[7]
		Public transit facility (B33)	[7]
		Shopping facility (B34)	[7]
		Fitness/recreation facility (B35)	[7]
		Medical institution (B36)	[7]
		Free public facility in/around the community (B37)	[7]
Perceived environment resource	Community management/service	Organizing activity (B38)	[5]
		Public safety (B39)	[7]
		Free public service (B40)	[8]

[1] Social Support Questionnaire

[2] RAND Social Health Battery

[3] Medical Outcomes Study Social Support Survey

[4] Duke-UNC Functional Social Support Questionnaire

[5] Social Adjustment Scale—Self-Report

[6] The Social Dysfunction Rating Scale

[7] The Neighborhood Environment Walkability Scale

[8] Satisfaction Scale for Community Nursing

### Reliability and validity assessments

The reliability and validity of the final versions were assessed after calculating the raw scores. The scoring method was the same as that in item selection.

#### Test-retest reliability

The time intervals between the test and re-test should be no longer than two weeks. A larger correlation coefficient indicated that the test-retest reliability of scale or dimensionality was better. Generally, if the correlation coefficient is larger than 0.80, then the correlation between two variables is desirable.

#### Internal consistency reliability

Cronbach’s α was used to assess the internal consistency of scale or dimensionality. In most cases, a satisfactory internal consistency indicates that the standardized Cronbach’s α coefficient is greater than 0.70 [31].

#### Inter-rater reliability

The McNemar-Bowker test was used to assess the agreement between two interviewers who had interviewed the same person. A good agreement meant that the weighted kappa was not less than 0.75 [32].

#### Concurrent validity

Firstly, the external criteria were those widely used in Chinese populations and had satisfactory reliability and validity. Any of the external criteria were used to assess just one of our dimensions because a comprehensive criterion of the SHSE does not exist. The correlation coefficient between the raw score of some dimension and the external criterion score should be statistically significant (*P* < 0.05). Additionally, the external criterion score should be comparatively low compared to the raw scores of unrelated dimensions or statistically unrelated (*P* ≥ 0.05).

#### Construct validity

A confirmatory factor analysis was performed to assess construct validity, and the maximum likelihood estimation was selected. If the goodness-of-fit index (GFI) and adjusted goodness-of-fit index (AGFI) were larger than 0.95 and 0.90, respectively, then the fitness of the model was desirable [33]. In addition, the root mean square error of approximation (RMSEA) can also be used to assess the degree of fit. If the RMSEA is less than 0.05, then the degree of fit is satisfactory; 0.05–0.08 indicates good fitness, and an RMSEA of less than 0.10 indicates moderate fitness [34].

#### Convergent and discriminant validity

The average variance extracted (AVE) of scale was calculated. If the AVE is larger than 0.50, then the convergent validity is good [35]. Discriminant validity is acceptable when the squared correlation coefficient of each two factors (factors were extracted when the eigenvalues were larger than 1 in the principal component analysis) was smaller than the AVE of the associated factors [36].

### Development of norms

The raw scores were calculated, and the T-test or Wilcoxon rank sum test was used to compare the distributions of the binary variables. Multiple categorical variables were compared using an analysis of variance or the Kruskal-Wallis H test. For better application of the SHSE, the standard norm and percentile rank norm were developed. The former can be applied when comparing two or more populations with different characteristics. The latter was easier for unprofessional people to understand, but the norm might not be descriptive for all Chinse elderly unless the sample was perfectly representative.

#### Standard norm

The equation for converting the raw score of some subject to the standard score (T score) was as follows: [37].

$$ {\mathrm{T}}_i=50+10\times \left({\mathrm{R}}_i-{\mathrm{M}}_{\mathrm{n}}\right)/{\mathrm{SD}}_{\mathrm{n}} $$

Where: T_*i*_ is the standard score of the subject; R_*i*_ is the raw score of the subject; M_n_ is the mean of the raw score; and SD_n_ is the standard deviation of the raw score.

#### Percentile rank norm

This norm showed the range of the raw score in each percentile rank [38].

## Results

### Phase 1: Development of draft scale

There were 3 dimensions, 9 sub-dimensions and 40 items in the revised draft scale (see Table 1). Only one item entitled “quality of natural environment” was added after consulting experts, and the other 39 items were selected from references. The questions and options and the scoring method of the items in the revised draft scale are shown in the Additional file 1. The raw score ranged between 40 and 200. A higher score represents a better social health status.

### Phase 2: Pilot testing and items selection

The pilot survey was performed from December 14, 2015 to January 8, 2016. Based on the ratio of subjects to items, the smallest sample size was 400. Considering the low response rates of similar surveys in China, the size of randomly drawing samples was nearly twice the minimum, and 271 potential participants refused to participate when community doctors approached them through telephone calls. Finally, 430 subjects were included in the statistical analysis, and 107 were interviewed twice. Six interviewees were excluded because of serious illness (physically or mentally disabled).

Table 2 shows the characteristics of the pilot test subjects. Mobility, self-care, daily activities, pain or discomfort, and anxiety or depression were the five dimensions in the European Quality of Life-5 Dimensions questionnaire assessed [39]. The “chronic diseases” in the variable “number of confirmed chronic diseases” included 12 diseases found in the top 10 lists of disease burden for the Chinese elderly [40]. The distributions of the two tests were significantly different regarding the type of household, religion, marital status and quality of sleep (*P < 0.05*). In addition, there were differences in mobility, daily activities, pain/discomfort and anxiety/depression between the pilot testing and field testing.

**Table 2:** Characteristics of the subjects in two tests

Characteristic	N (%)		*P*-value^#^
	Pilot testing	Field testing	
Demographic and socio-economic variables			
Gender			0.63
Male	215 (50.00)	1172 (48.75)	
Female	215 (50.00)	1232 (51.25)	
Age group			0.19
60~ 69 years	231 (53.72)	1213 (50.46)	
70~ 79 years	140 (32.56)	818 (34.03)	
80 years and above	59 (13.72)	373 (15.52)	
Type of household			< 0.01^a^
Agricultural	22 (5.12)	664 (27.62)	
Non-agricultural	257 (59.77)	1105 (45.97)	
Agricultural to non-agricultural	151 (35.12)	617 (25.67)	
Others	0 (0.00)	18 (0.75)	
Religion			< 0.01^b^
Buddhism	130 (30.23)	831 (34.63)	
Christianity	5 (1.16)	134 (5.58)	
Others	0 (0)	14 (0.58)	
None	295 (68.60)	1421 (59.21)	
Education level			0.15
Uneducated	96 (22.33)	545 (22.71)	
Did not completed primary school	88 (20.47)	417 (17.38)	
Primary school graduate	64 (14.88)	493 (20.54)	
Middle school graduate	93 (21.63)	570 (23.75)	
High school graduate	40 (9.30)	241 (10.04)	
College/university graduate	49 (11.40)	134 (5.58)	
Marital status			0.02^c^
Never married	0 (0)	7 (0.29)	
Married	328 (76.28)	1933 (80.54)	
Divorced	11 (2.56)	20 (0.83)	
Widowed	91 (21.16)	440 (18.33)	
Behavioral variables			
Living alone			0.62
Yes	38 (8.84)	229 (9.59)	
No	392 (91.16)	2159 (90.41)	
Quality of sleeping			< 0.01
Sleep very well all the time	232 (53.95)	583 (24.27)	
Sleep well in most of the time	139(32.33)	1300 (54.12)	
Sleep badly in most of the time	38 (8.84)	414 (17.24)	
Sleep very badly all the time	21 (4.88)	105 (4.37)	
Current smoker			0.66
Yes	73 (16.98)	429 (17.85)	
No	357 (83.02)	1974 (82.15)	
Current drinker			0.83
Yes	97 (22.56)	554 (23.04)	
No	333 (77.44)	1850 (76.96)	
Health status variables			
Mobility^*^			< 0.01^d^
Have no problems in walking about	355 (82.56)	2201 (91.56)	
Have some problems in walking about	72 (16.74)	193 (8.03)	
Be confined to bed	3 (0.70)	10 (0.42)	
Self-care^*^			0.98^e^
Have no problems with self-care	419 (97.44)	2342 (97.46)	
Have some problems in washing/dressing	8 (1.86)	53 (2.21)	
Be unable to wash/dress	3 (0.70)	8 (0.33)	
Usual activities^*^			< 0.01^f^
Have no problems with performing	381 (88.60)	2246 (93.47)	
Have some problems with performing	46 (10.70)	139 (5.78)	
Be unable to perform	3 (0.70)	18 (0.75)	
Pain/discomfort^*^			< 0.01
Have no pain/discomfort	243 (56.51)	1533 (63.77)	
Have moderate pain/discomfort	162 (37.67)	804 (33.44)	
Have extreme pain/discomfort	25 (5.81)	67 (2.79)	
Anxiety/depression^*^			0.02^g^
Be not anxious/depressed	396 (92.09)	2119 (88.37)	
Be moderately anxious/depressed	30 (6.98)	262 (10.93)	
Be extremely anxious/depressed	4 (0.93)	17 (0.71)	
Number of confirmed chronic diseases			0.86
0	104 (24.19)	608 (25.29)	
1	146 (33.95)	792 (32.95)	
2	113 (26.28)	577 (24.00)	
3~	67 (15.58)	427 (17.76)	

^*^ The dimension of the three-level EuroQol five-dimensional questionnaire

^#^*P*-value of Chi-square test (the difference of distribution between two tests)

^a^ Combined “agricultural to non-agricultural” with “others” before Chi-square test

^b^ Combined “Christianity” with “others” before Chi-square test

^c^ Combined “never married” with “divorced” before Chi-square test

^d^ Combined “have some problems in walking about” with “be confined to bed” before Chi-square test

^e^ Combined “have some problems in washing/dressing” with “be unable to wash/dress” before Chi-square test

^f^ Combined “have some problems with performing” with “be unable to perform” before Chi-square test

^g^ Combined “be moderately anxious/depressed” with “be extremely anxious/depressed” before Chi-square test

Based on 5 different statistical methods, the items in the revised draft scale were extracted. The items in the final versions of the SHSE are shown in Table 3. There were 25 items in the SHSE-L and 14 items in the SHSE-S.

**Table 3:** Items in the Social Health Scale for the Elderly after selection

Variable	Correlation coefficient ^[1]^	Standardized Cronbach’s α ^[2]^	Correlation coefficient^[3]^	Z value^[4]^	Factor loading ^[5]^	Results (“√” = be retained)	
						Long form ^[6]^	Short form ^[7]^
B01	0.2951	0.7717^*^	0.4362^*^	6.5122^*^	0.4777^*^	√	
B02	0.1501	0.7670^*^	0.5771^*^	7.6243^*^	0.6941^*^	√	
B03	0.1050	0.7638^*^	0.6478^*^	8.8509^*^	0.6898^*^	√	
B04	0.3028^*^	0.7638^*^	0.6636^*^	9.3755^*^	0.6358^*^	√	√
B05	0.4883^*^	0.7634^*^	0.6433^*^	8.8892^*^	0.5528^*^	√	√
B06	0.1993	0.7625^*^	0.6239^*^	9.8041^*^	0.6058^*^	√	
B07	0.3132^*^	0.7669^*^	0.5304^*^	7.7367^*^	0.5998^*^	√	√
B08	0.3704^*^	0.7689^*^	0.3908	6.6259^*^	0.3958		
B09	0.1248	0.7619^*^	0.5238^*^	9.6481^*^	0.4996^*^	√	
B10	0.2145	0.7687^*^	0.4658^*^	7.2698^*^	0.6026^*^	√	
B11	0.1076	0.7659^*^	0.5189^*^	8.3519^*^	0.6479^*^	√	
B12	0.4287^*^	0.7719^*^	0.4605^*^	5.6679^*^	0.4910^*^	√	√
B13	0.5114^*^	0.7754^*^	0.2266	3.0738^*^	0.6257^*^	√	
B14	0.1537	0.7701^*^	0.3443	5.5538^*^	0.5017^*^		
B15	0.5956^*^	0.7839	0.2181	0.6658	0.6637^*^		
B16	0.3743^*^	0.7833	0.1110	1.8150	0.6253^*^		
B17	0.6986^*^	0.7752^*^	0.4340^*^	4.3566^*^	0.1904	√	
B18	0.3985^*^	0.7788	0.2065	3.1371^*^	0.3861		
B19	0.3729^*^	0.7690^*^	0.5217^*^	7.7453^*^	0.5102^*^	√	√
B20	0.4263^*^	0.7704^*^	0.4233^*^	6.2262^*^	0.4694^*^	√	√
B21	0.5608^*^	0.7727^*^	0.5755^*^	7.0495^*^	0.6480^*^	√	√
B22	0.3586^*^	0.7732^*^	0.3726	5.0186^*^	0.6386^*^	√	
B23	0.4078^*^	0.7747^*^	0.2380	3.6880^*^	0.3728		
B24	0.4036^*^	0.7701^*^	0.3664	7.6728^*^	0.3398		
B25	−0.0693	0.7817	0.0964	0.6503	0.6128^*^		
B26	0.0981	0.7811	0.0736	1.5413	0.6299^*^		
B27	0.1912	0.7745^*^	0.3755	4.6316^*^	0.6877^*^		
B28	0.5721^*^	0.7750^*^	0.4149^*^	4.8409^*^	0.4470^*^	√	√
B29	0.4121^*^	0.7733^*^	0.3727	5.1077^*^	0.3892		
B30	0.1868	0.7717^*^	0.3145	5.1113^*^	0.4890^*^		
B31	0.3664^*^	0.7795	0.2044	− 0.7260	0.4446^*^		
B32	0.3289^*^	0.7756^*^	0.4758^*^	3.2981^*^	0.4370^*^	√	√
B33	0.6003^*^	0.7696^*^	0.5738^*^	6.2249^*^	0.5290^*^	√	√
B34	0.1267	0.7738^*^	0.3069	3.2000^*^	0.7324^*^		
B35	0.5312^*^	0.7725^*^	0.5139^*^	5.6521^*^	0.6615^*^	√	√
B36	0.4733^*^	0.7765^*^	0.4241^*^	3.8774^*^	0.6434^*^	√	√
B37	0.2295	0.7731^*^	0.4523^*^	5.1986^*^	0.6925^*^	√	
B38	0.5854^*^	0.7757^*^	0.4700^*^	3.9140^*^	0.6335^*^	√	√
B39	0.3592^*^	0.7848	0.1114	− 1.2338	0.4136^*^		
B40	0.5565^*^	0.7689^*^	0.5480^*^	6.7172^*^	0.5877^*^	√	√

*N* = 430

^*^ The related item was appropriate to be retained after analyzing by one of selection method

^[1]^ Test-retest reliability analysis: if the Spearman rank correlation coefficient was larger than 0.30 (*P* < 0.05), then the related item was appropriate to be retained

^[2]^ Cronbach’s alpha analysis: If the standardized Cronbach’s α of scale was less than that after eliminating some item, than the related item was not appropriate to be retained

^[3]^ Correlation analysis: Comparing the score of some item with that of dimension it belonged to. If the Spearman rank correlation coefficient was larger than 0.40 (P < 0.05), then the related item was appropriate to be retained

^[4]^ Distinguishability analysis: Comparing the raw scores of some item between the high-score group of scale (P_75_) and low-score group of scale (P_25_). If the *P*-value of Wilcoxon rank sum test was less than 0.05, then the related item was appropriate to be retained

^[5]^ Factor analysis: If the factor loading of some item was larger than 0.40, then the related item was appropriate to be retained

^[6]^ If less than 2 selection methods indicated that some item was not appropriate to be retained, than the related item would be retain in the long form

^[7]^ If each selection method indicated that some item was appropriate to be retained, than the related item would be retain in the short form

### Phase 3: Field testing, reliability and validity assessments

The field testing was performed from November 6, 2016 to January 20, 2017. A total of 2415 residents were interviewed, and 11 of them were excluded before the statistical analysis because of missing data in the SHSE. In total, 494 subjects were interviewed twice. The differences between the distributions of subjects in the two tests were not statistically significant for gender, age group, education level, the status of living alone, smoking status, drinking status, the ability of self-care, or the number of confirmed chronic diseases (see Table 2).

#### Test-retest reliability

The correlations (Spearman’s correlation analysis) of any two items in the SHSE-L ranged from 0.41 to 0.87. The correlations of scales were 0.77 (SHSE-L) and 0.78 (SHSE-S). In the SHSE-L, the correlations of dimensions were 0.61 (SS), 0.81 (SA) and 0.78 (PER), and those correlations were 0.49, 0.79 and 0.78 in the SHSE-S, respectively. Each correlation was statistically significant.

#### Internal consistency reliability

In terms of the SHSE-L, the standardized Cronbach’s α coefficient of scale was 0.79, and those of dimensions were 0.85 (SS), 0.61 (SA) and 0.65 (PER). With regard to the SHSE-S, the standardized Cronbach’s α coefficient of scale was 0.65, and those of dimensions were 0.69 (SS), 0.55 (SA) and 0.63 (PER).

#### Inter-rater reliability

In total, 43.12% of the subjects who were interviewed twice were interviewed by different interviewers. Both the McNemar-Bowker tests (SHSE-L and SHSE-S) indicated disagreement between the interviewers (*P* < 0.01). The weighted kappas were 0.44 (SHSE-L) and 0.43 (SHSE-S).

#### Concurrent validity

The Social Support Rate Scale (SSRS) has been widely used to assess social support of the Chinese [41], and it was selected as the external criterion of SS. One question used to assess the relationship between the interviewee and his or her colleagues was removed, so the maximum aggregate score was 62. A total of 2358 subjects did not have missing data in the SSRS. Spearman’s correlation analyses were conducted to assess the correlations between SSRS and SS, SA, or PER. Moderate correlations were identified between the SSRS and SS parts of the SHSE-L and SHSE-S. The correlations between the SSRS and SS were 0.64 (*P* < 0.01) and 0.61 (*P* < 0.01) in the SHSE-L and SHSE-S, respectively. In addition, the SSRS was uncorrelated or weakly correlated with SA and PER in both the SHSE-L (SA: *r* = 0.23, *P* < 0.01; PER: *r* = 0.03, *P* > 0.05) and the SHSE-S (SA: *r* = 0.20, *P* < 0.01; PER: *r* = 0.01, *P* > 0.05).

#### Construct validity

**Fig. 1:**
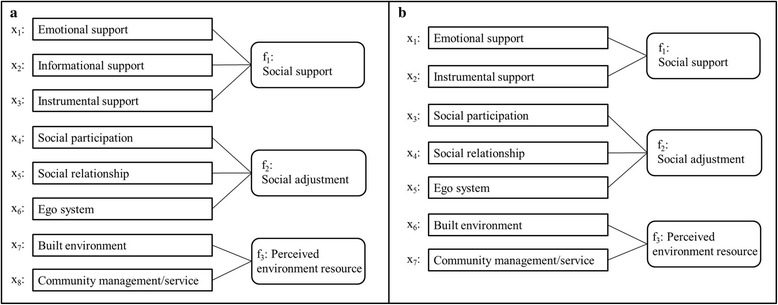
The structures of Model I (**a**) and model II (**b**) in confirmatory factor analysis

Two models were constructed, one based on the SHSE-L (model I) and another based on the SHSE-S (model II). Model I was listed as follows: x_1_ = a_1_*f_1_ + e_1_, x_2_ = a_2_*f_1_ + e_2_, x_3_ = a_3_*f_1_ + e_3_, x_4_ = a_4_*f_2_ + e_4_, x_5_ = a_5_*f_2_ + e_5_, x_6_ = a_6_*f_2_ + e_6_, x_7_ = a_7_*f_3_ + e_7_, x_8_ = a_8_*f_3_ + e_8_. Model II was listed as follows: x_1_ = a_1_*f_1_ + e_1_, x_2_ = a_2_*f_1_ + e_2_, x_3_ = a_3_*f_2_ + e_3_, x_4_ = a_4_*f_2_ + e_4_, x_5_ = a_5_*f_2_ + e_5_, x_6_ = a_6_*f_3_ + e_6_, x_7_ = a_7_*f_3_ + e_7_. In the equations, a_i_ and e_i_ represent coefficients and x_i_ and f_i_ represent sub-dimensions and dimensions, respectively. Figure 1 shows the relationships between sub-dimensions (x_i_) and dimensions (f_i_) in the two models. In model I, GFI = 0.95, AGFI = 0.90, and RMSEA = 0.10. In model II, GFI = 0.97, AGFI = 0.93, and RMSEA = 0.09.

#### Convergent and discriminant validity

The AVEs of the SHSE-L and SHSE-S were 0.54 and 0.53, respectively. Table 4 shows the matrix of factor loadings after being rotated by Varimax in the principal component analysis. Six and four factors were extracted in the principal component analysis of the SHSE-L and SHSE-S, respectively. The AVEs of every two factors were larger than the squared correlation coefficients of related factors in both versions of the SHSE (SHSE-L: the AVEs of the factors ranged from 0.31 to 0.78, and the maximum squared correlation coefficient was 0.14; SHSE-S: the AVEs of the factors ranged from 0.33 to 0.66, and the maximum squared correlation coefficient was 0.10).

**Table 4:** The matrix of factor loadings after being rotated by Varimax

Variables	Long form						Short form			
	Factor1	Factor2	Factor3	Factor4	Factor5	Factor6	Factor1	Factor2	Factor3	Factor4
B01	0.50									
B02		0.39								
B03		0.92								
B04		0.92					0.45			
B05	0.83						0.88			
B06		0.46								
B07	0.87						0.87			
B09	0.58									
B10						0.47				
B11	0.57									
B12	0.56						0.61			
B13	0.74									
B17						−0.57				
B19				0.54				0.49		
B20			0.65					0.58		
B21			0.71					0.72		
B22			0.67							
B28			0.47					0.65		
B32				0.57					0.48	
B33					0.74					0.75
B35					0.50				0.54	
B36					0.76					0.80
B37				0.69						
B38				0.63					0.75	
B40				0.54					0.63	

### Phase 4: Development of norms

Table 5 shows the distributions of raw scores in the field testing. Except for the status of living alone and the number of confirmed chronic diseases, the distributions of the other variables were similar between the SHSE-L and SHSE-S. The differences were statistically significant for gender, age group, type of household, religion, education level, marital status, quality of sleeping, smoking status, the ability of mobility, the ability of self-care, the ability of daily activities, and anxiety status. Female, young elderly, Christian, highly educated, and married persons had better social health. Living alone; poor quality of sleep; current smoking; poor ability of mobility, self-care and daily activities; and serious anxiety/depression might imply worse social health. The standard norm and percentile rank norm are shown in the Additional file 1. Generally, SS and SA changed with age, so the same norm was not suitable for every age group. Taking these results into consideration, we generated three different norms for the three age groups.

**Table 5:** The distribution of raw score of the Social Health Scale for the Elderly

Characteristic	Long form		Short form	
	Median (QR)	*P*-value	Median (QR)	*P*-value
Demographic and socio-economic variables				
Gender		< 0.01^*^		< 0.01^*^
Male	62.00 (13.00)		37.00 (9.00)	
Female	64.00 (14.00)		38.00 (9.00)	
Age group		< 0.01^#^		< 0.01^#^
60~ 69 years	65.00 (14.00)		39.00 (10.00)	
70~ 79 years	62.00 (13.00)		38.00 (9.00)	
80 years and above	58.00 (13.00)		35.00 (9.00)	
Type of household		< 0.01^#^		< 0.01^#^
Agricultural	61.00 (14.00)		34.00 (9.00)	
Non-agricultural	63.00 (14.00)		40.00 (8.00)	
Agricultural to non-agricultural	64.00 (13.00)		38.00 (8.00)	
Others	65.00 (16.00)		42.00 (12.00)	
Religion		< 0.01^#^		< 0.01^#^
Buddhism	63.00 (14.00)		37.00 (8.00)	
Christianity	58.50 (15.00)		35.00 (9.00)	
Others	67.50 (10.00)		39.00 (9.00)	
None	63.00 (13.00)		39.00 (9.00)	
Education level		< 0.01^#^		< 0.01^#^
Uneducated	61.00 (13.00)		35.00 (7.00)	
Did not completed primary school	61.00 (15.00)		36.00 (9.00)	
Primary school graduate	63.00 (13.00)		37.00 (8.00)	
Middle school graduate	65.00 (13.00)		40.00 (9.00)	
High school graduate	65.00 (13.00)		41.00 (9.00)	
College/university graduate	67.50 (12.00)		42.00 (9.00)	
Marital status		< 0.01^#^		< 0.01^#^
Never married	56.00 (10.00)		35.00 (4.00)	
Married	64.00 (13.00)		39.00 (9.00)	
Divorced	59.00 (9.50)		37.00 (7.00)	
Widowed	59.00 (12.00)		36.00 (8.00)	
Behavioral variables				
Living alone		< 0.01^*^		0.20^*^
Yes	60.00 (14.00)		37.00 (9.00)	
No	63.00 (13.00)		38.00 (10.00)	
Quality of sleeping		< 0.01^#^		< 0.01^#^
Sleep very well all the time	65.00 (13.00)		39.00 (10.00)	
Sleep well in most of the time	63.00 (13.50)		37.00 (9.00)	
Sleep badly in most of the time	63.00 (14.00)		38.00 (10.00)	
Sleep very badly all the time	58.00 (12.00)		36.00 (10.00)	
Current smoker		0.03^*^		< 0.01^*^
Yes	62.00 (14.00)		37.00 (9.00)	
No	63.00 (13.00)		38.00 (10.00)	
Current drinker		0.77^*^		0.17^*^
Yes	63.00 (12.00)		38.00 (9.00)	
No	63.00 (14.00)		38.00 (10.00)	
Health status variables				
Mobility ^a^		< 0.01^#^		< 0.01^#^
Have no problems in walking about	63.00 (13.00)		38.00 (9.00)	
Have some problems in walking about	59.00 (15.00)		35.00 (9.00)	
Be confined to bed	44.00 (20.00)		25.50 (10.00)	
Self-care ^a^		< 0.01^#^		< 0.01^#^
Have no problems with self-care	63.00 (13.00)		38.00 (10.00)	
Have some problems in washing/dressing	57.00 (16.00)		34.00 (10.00)	
Be unable to wash/dress	47.00 (15.50)		26.00 (7.50)	
Usual activities ^a^		< 0.01^#^		< 0.01^#^
Have no problems with performing	63.00 (13.00)		38.00 (9.00)	
Have some problems with performing	58.00 (14.00)		34.00 (8.00)	
Be unable to perform	50.00 (14.00)		28.50 (12.00)	
Pain/discomfort ^a^		0.92^#^		0.34^#^
Have no pain/discomfort	63.00 (14.00)		38.00 (9.00)	
Have moderate pain/discomfort	63.00 (12.00)		38.00 (8.00)	
Have extreme pain/discomfort	62.00 (19.00)		39.00 (11.00)	
Anxiety/depression ^a^		< 0.01^#^		< 0.01^#^
Be not anxious/depressed	63.00 (13.00)		38.00 (10.00)	
Be moderately anxious/depressed	61.00 (13.00)		37.00 (8.00)	
Be extremely anxious/depressed	59.00 (15.00)		36.00 (8.00)	
Number of confirmed chronic diseases		0.61^#^		< 0.01^#^
0	63.00 (13.00)		37.00 (9.00)	
1	63.00 (14.00)		38.00 (9.00)	
2	63.00 (14.00)		38.00 (9.00)	
3~	62.00 (15.00)		39.00 (9.00)	

*N* = 2404

^*^P-value of Wilcoxon rank sum test

^#^*P*-value of Kruskal-Wallis H test

^a^ The dimension of the three-level EuroQol five-dimensional questionnaire

## Discussion

This study developed two versions of the SHSE, with 25 items in the long form and 14 items in the short form. Each form could assess three dimesons of social health, and both social health of the individual and social health of society were measured. The reliability and validity of the two versions were acceptable. Two norms could reflect the social health status of the generally healthy elderly living in Hangzhou. We believe that the SHSE-L can be used to explore the risk or protective factors of social health, and the SHSE-S can be combined with other domains of health status (e.g., mental health) to assess comprehensive health status. Usually, the short forms of scales are generated based on their longer forms, such as the SF-12 [42]; therefore, we suggest further studies for the development of the SHSE-S, although the reliability and validity results of the SHSE-S were similar to those of the SHSE-L.

This study had the following limitations: firstly, the response rate of the pilot testing was not very good [43], so non-respondent bias existed. Neither random sampling survey nor census was performed during the field testing. Compared to the pilot testing sample, some differences were present (Table 2); thus, the representativeness of the field testing sample was not desirable, and volunteer bias was inevitable. All the participants lived in Hangzhou; therefore, the representativeness of the sample was not satisfactory. Secondly, the test-retest reliability and inter-rater reliability of the SHSE-L and SHSE-S were acceptable but were far from perfect. The internal consistency of the SHSE-S was lower than the optimum level. All of the above limitations might result from imperfect design of the questions and options. Because of the lack of applicative external criteria about the SHSE, SA and PER, the concurrent validity assessment was not completed. Thirdly, the application of SHSE was not wide enough because of the lack of multiple cultures in the stage of developing the draft scale; therefore, Chinese elderly who live in different cultures might not be suitable for assessment with this scale. Finally, this study lacked a comprehensive outcome variable to assess the contribution of social health to the comprehensive health status of the elderly.

The social adjustments of people in different cultures are diverse [44]. China is a multi-ethnic society; therefore, the existence of multi-cultures is inevitable in China. Similarly, the levels of SS and PER might also be diversified. It was difficult to generate a scale/norm that could be applied universally in China based on one study. For better utility, the validity and reliability of the SHSE-L and SHSE-S should be assessed based on a representative sample or total population. Then, the SHSE-L and SHSE-S should be revised to improve their reliability and validity. Finally, the norms of the SHSE-L and SHSE-S could be widely used in the assessment of social health status of all Chinese elderly.

Previous studies have indicated that the agreement of answers between scales designed for self-report and scales designed for short interviews are not optimistic [45]. Therefore, we do not suggest that residents complete the SHSE-L or SHSE-S by themselves; rather, we recommend that trained personnel complete the scales by interviewing the participants. Additionally, there were some problems with the interviewers, such as improper ways of asking sensitive questions, time and site constraints, and interviewer bias. Self-report versions of the SHSE-L and SHSE-S should be generated in the future.

## Conclusion

For successful ageing, a suitable instrument to measure health status is necessary. This study developed a long and short form of the SHSE (SHSE-L and SHSE-S, respectively) to measure the social health status of the Chinese elderly, which fills a gap in social health assessment. The standard norms and percentile rank norms of the social health of the elderly in Hangzhou city were generated, which can be used as references in other studies.

## Additional file

Additional file 1: The draft, scoring method and norms of the Social Health Scale for the Elderly. (DOCX 46 kb)

## Abbreviations

AGFI: Adjusted goodness-of-fit index
AVE: Average variance extracted
GFI: Goodness-of-fit index
PER: Perceived environment resource
RMSEA: Root mean square error of approximation
SA: Social adjustment
SHSE: Social Health Scale for the Elderly
SHSE-L: Long form of the Social Health Scale for the Elderly
SHSE-S: Short form of the Social Health Scale for the Elderly
SS: Social support
SSRS: Social Support Rate Scale
WHO: World Health Organization
