# Supplementary material for: The development of the social health scale for the elderly
Source: Health Qual Life Outcomes. 2018 Apr 18;16:67. doi: 10.1186/s12955-018-0899-6 (PMC5907369; doi:10.1186/s12955-018-0899-6)
Supplement: Supplementary file 1 — The draft, scoring method and norms of the Social Health Scale for the Elderly. (DOCX 46 kb) [file 12955_2018_899_MOESM1_ESM.docx]

**The Social Health Scale for the Elderly (draft)**

**The options of question B01-B14:**

[1] = None

[2] = Children (natural/adopted children);

[3] = Partner (spouse/a person of the opposite sex in a permanent relationship);

[4] = Friends;

[5] = Other relatives (blood relatives or close relatives by marriage excluding natural children and spouse)

[6] = Others

***Question B01-B15 can have multiple answers. Please draw a circle on the option.***

**B01** Who can be a listener when you want to pour out your heart? [1] [2] [3] [4] [5] [6]

**B02** Who accepts you totally, including your worst points? [1] [2] [3] [4] [5] [6]

**B03** Who can understand your problems/ideas? [1] [2] [3] [4] [5] [6]

**B04** Who can support you in major decisions you make? [1] [2] [3] [4] [5] [6]

**B05** Who can care about you, regardless of what is happening to you? [1] [2] [3] [4] [5] [6]

**B06** Who can accompany you to pass the time? [1] [2] [3] [4] [5] [6]

**B07** Who can comfort you when you need it? [1] [2] [3] [4] [5] [6]

**B08** Who can make you moved? [1] [2] [3] [4] [5] [6]

**B09** Who can give you information to help you understand a situation? [1] [2] [3] [4] [5] [6]

**B10** Who can tell you where you need to improve in a thoughtful manner? [1] [2] [3] [4] [5] [6]

**B11** Who can give you useful suggestions that help you to make decisions? [1] [2] [3] [4] [5] [6]

**B12** Who can help you with daily chores if you were sick? [1] [2] [3] [4] [5] [6]

**B13** Who can give you financial aid? [1] [2] [3] [4] [5] [6]

**B14** Who can give you material aid? [1] [2] [3] [4] [5] [6]

**B15** Which of the following house works you are helped with in daily life?

[1] None [2] Cooking [3] Purchasing [4] Cleaning the room

[5] Folding laundry [6] Washing clothes [7] Others

**The options of question B16-B22:**

[1] = Less than once every three months;

[2] = Once/more than once every three months, but less than once every month;

[3] = One to four times every month;

[4] = Two to four times every week;

[5] = More than four times every week;

***Question B16-B24 have single answers. Please draw a circle on the option.***

**B16** How often did you work for pay in the last year? [1] [2] [3] [4] [5]

**B17** How often did you do housework in the last year? [1] [2] [3] [4] [5]

**B18** How often did you do volunteer work in the last year? [1] [2] [3] [4] [5]

**B19** How often did you participate in collective recreational activities in the last year? [1] [2] [3] [4] [5]

***The definitions of children, other relatives and partner in question B20, B22 and B24 are like above.***

**B20** How often did you chat with children for a long time in the last year? [1] [2] [3] [4] [5]

**B21** How often did you chat with friends for a long time in the last year? [1] [2] [3] [4] [5]

**B22** How often did you chat with other relatives in the last year? [1] [2] [3] [4] [5]

**B23** How about the relationship with your neighbors in the last two weeks?

[1] Having quarreled with each other more than twice

[2] Having quarreled with each other once/twice

[3] Having never chatted with each other

[4] Having greeted with each other when you encountered

[5] Having gone to each other’s house

**B24** How about the relationship with your partner in the last two weeks?

[1] Having quarreled with each other more than twice

[2] Having quarreled with each other once/twice

[3] Neither having quarreled with each other nor having had a close relationship

[4] Having had a close relationship most of the time

[5] Having had a close relationship all the time

***Question B25 can have multiple answers. Please draw a circle on the option.***

**B25** Which of the following psychological states you have ever had in social contact?

[1] None [2] Manipulation [3] Suspiciousness [4] Over-dependency

[5] Nervousness [6] Disappointment [7] Hostility [8] Emotional withdrawal

**The options of question B26-B30:**

[1] = Almost never

[2] = Once in a while

[3] = About half the time

[4] = Most of the time

[5] = Always

***Question B26-B30 have single answers. Please draw a circle on the option.***

**B26** How much time have you felt affection toward others in the last year? [1] [2] [3] [4] [5]

**B27** How much time have you concerned for self-health in the last year? [1] [2] [3] [4] [5]

**B28** How much spare time have you spent on hobbies or interests in the last year? [1] [2] [3] [4] [5]

**B29** How much time have your family financial been secured in the last year? [1] [2] [3] [4] [5]

**B30** How much time have you been optimistic in the last year? [1] [2] [3] [4] [5]

***Question B31-B32 can have multiple answers. Please draw a circle on the option.***

**B31** Which of the following kinds of physical environment were bad more than half of the time in the last year?

[1] None [2] Domestic water [3] Noise [4] Public health [5] Air

**B32** Which of the following manufactured landscapes have been seen in/around the community you lived in the last year?

[1] None [2] Artificial hill [3] Artificial lake and pond

[4] Artificial lawn [5] Garden building [6] Artificial planting flowers and trees

***Question B33-B36 have single answers. Please write the answer on the line.***

**B33** If you go to the nearest public transit facility (bus stop/subway station/bicycle rental station) from your house by , you should spend minutes.

[1] Walk [2] Bicycle [3] Electric bicycle [4] Bus [5] Private car

**B34** If you go to the nearest shopping facility (farmer’s market/supermarket/convenience store/retail store) from your house by , you should spend minutes.

[1] Walk [2] Bicycle [3] Electric bicycle [4] Bus [5] Private car

**B35** If you go to the nearest fitness/recreation facility (outdoor exercise machine/gymnasium/university for the elderly/community entertainment room) from your house by , you should spend minutes.

[1] Walk [2] Bicycle [3] Electric bicycle [4] Bus [5] Private car

**B36** If you go to the nearest medical institution (general hospital/special hospital/community health service center /township hospital/village clinic/private clinic) from your house by , you should spend minutes.

[1] Walk [2] Bicycle [3] Electric bicycle [4] Bus [5] Private car

***Question B37 can have multiple answers. Please draw a circle on the option.***

**B37** Which of the following public facilities have been seen in/around the community you lived in the last year?

[1] None [2] Footpath [3] Bicycle lane [4] Special entrance for the disabled [5] Free parking lot

[6] Free playground/garden [7] Free swimming pool [8] Free stadium [9] Dumping site

***Question B38 has single answer. Please draw a circle on the option.***

**B38** How many times has the community you lived in organized the activities in the last year?

[1] Never [2] Once or twice [3] Three or four times [4] Five or six times [5] More than six times

***Question B39-B40 can have multiple answers. Please draw a circle on the option.***

**B39** Which of the following situations/events have occurred in/around the community you lived in the last year?

[1] None

[2] The lamplight was dim or broke down for a long time

[3] Public security door/monitor broke down for a long time

[4] Someone was attacked by stray animals

[5] The security personnel was lacking responsibility in his work

[6] Crime incident

**B40** Which of the following free public services have been offered by the community you lived in in the last year?

[1] None

[2] Door-to-door medical service

[3] Chatting with the oldest old, the disabled/ bedridden/solitary elderly

[4] Handling the dispute between neighbors

[5] Health education

[6] Legal aid/advice

[7] Distributing supply

[8] Nursing the oldest old, the disabled/ bedridden/solitary elderly

**The scoring method of items in the Social Health Scale for the Elderly (draft)**

| Variables | Recording rule^#^ | | | | |
| --- | --- | --- | --- | --- | --- |
| B01-B15, B32 | [1] = 1 | 1 = 2 | 2 = 3 | 3 = 4 | 4 or more= 5 |
| B16-B24, B26-B30, B38 | [1] = 1 | [2] = 2 | [3] = 3 | [4] = 4 | [5] = 5 |
| B25, B31, B39 | 4 or more = 1 | 3 = 2 | 2 = 3 | 1 = 4 | [1] = 5 |
| B33-B36^*^ | 31^a^ or more = 1^b^ | 21^a^ to 30^a^ = 2 | 11^a^ to 20^a^ = 3 | 6^a^ to 10^a^ = 4 | 5^a^ or less = 5 |
| B37, B40 | [1] = 1 | 1, 2 = 2 | 3, 4 = 3 | 5, 6 = 4 | 7 or higher = 5 |
| Note  ^#^ The left of equal sign: the number of selected option excluding “none” (Arabic numerals)/option (parenthesized Arabic numerals)/calculation (superscripted Arabic numerals). The right of equal sign means the raw score of each variable.  ^*^ Speed conversion equation (parenthesized Arabic numerals mean options): [2] = [1]*3, [3] = [1]*6, [4] = [1]*6, [5] = [1]*12.  ^a^ Calculation: the length of walking time (minutes) after converting based on the speed conversion equation.  ^b^ If the length of time cannot been estimated because the interviewee has never gone to such site, then this score will be chosen. | | | | | |

**The standard norms of the Social Health Scale for the Elderly**

| T score | Raw score | | | | | | | |
| --- | --- | --- | --- | --- | --- | --- | --- | --- |
|  | Long form | | | | Short form | | | |
|  | 60-69 years | 70-79 years | 80+ years | Total | 60-69 years | 70-79 years | 80+ years | Total |
| 20 |  |  |  |  |  | 18 |  |  |
| 21 |  |  |  |  |  |  |  | 18 |
| 22 |  |  |  |  |  |  |  |  |
| 23 |  |  |  |  |  |  |  |  |
| 24 |  |  |  | 35 | 20 |  |  | 20 |
| 25 |  | 37 |  |  |  |  | 20 |  |
| 26 |  | 38 |  | 37 | 21 | 22 |  | 21 |
| 27 | 39 |  | 35 | 38 | 22 | 23 |  | 22 |
| 28 | 40-41 | 40 |  | 39 | 23 |  |  | 23 |
| 29 | 42 | 41 |  | 40 |  | 24 | 22 |  |
| 30 | 43 | 42 | 38 | 41-42 | 24 | 25 | 23 | 24 |
| 31 | 44 | 43 |  | 43 | 25 |  |  | 25 |
| 32 | 45 | 44-45 | 40-41 | 44 | 26 | 26 | 24 |  |
| 33 | 46 | 46 | 42 | 45 |  | 27 | 25 | 26 |
| 34 | 47 | 47 | 43 | 46 | 27 |  |  | 27 |
| 35 | 48-49 | 48 | 44 | 47 | 28 | 28 | 26 |  |
| 36 | 50 | 49 | 45 | 48 | 29 | 29 | 27 | 28 |
| 37 | 51 | 50 | 46 | 49 |  |  |  | 29 |
| 38 | 52 | 51 | 47 | 50 | 30 | 30 | 28 | 30 |
| 39 | 53 | 52 | 48 | 51-52 | 31 | 31 | 29 |  |
| 40 | 54 | 53 | 49 | 53 |  |  |  | 31 |
| 41 | 55 | 54 | 50 | 54 | 32 | 32 | 30 | 32 |
| 42 | 56 | 55 | 51 | 55 | 33 | 33 |  |  |
| 43 | 57-58 | 56 | 52 | 56 | 34 |  | 31 | 33 |
| 44 | 59 | 57 | 53 | 57 |  | 34 | 32 | 34 |
| 45 | 60 | 58 | 54 | 58 | 35 |  |  |  |
| 46 | 61 | 59 | 55 | 59 | 36 | 35 | 33 | 35 |
| 47 | 62 | 60 | 56 | 60 | 37 | 36 | 34 | 36 |
| 48 | 63 | 61 | 57 | 61 |  |  |  | 37 |
| 49 | 64 | 62 | 58 | 62-63 | 38 | 37 | 35 |  |
| 50 | 65-66 | 63 | 59 | 64 | 39 | 38 | 36 | 38 |
| 51 | 67 | 64 | 60 | 65 |  |  |  | 39 |
| 52 | 68 | 65 | 61 | 66 | 40 | 39 | 37 |  |
| 53 | 69 | 66 | 62 | 67 | 41 | 40 | 38 | 40 |
| 54 | 70 | 67 | 63 | 68 | 42 |  |  | 41 |
| 55 | 71 | 68 | 64 | 69 |  | 41 | 39 |  |
| 56 | 72 | 69 | 65 | 70 | 43 | 42 |  | 42 |
| 57 | 73 | 70 | 66 | 71 | 44 |  | 40 | 43 |
| 58 | 74-75 | 71 | 67 | 72-73 | 45 | 43 | 41 | 44 |
| 59 | 76 | 72 | 68 | 74 |  | 44 |  |  |
| 60 | 77 | 73 | 69 | 75 | 46 |  | 42 | 45 |
| 61 | 78 | 74-75 | 70 | 76 | 47 | 45 | 43 | 46 |
| 62 | 79 | 76 | 71 | 77 |  | 46 |  |  |
| 63 | 80 | 77 | 72 | 78 | 48 |  | 44 | 47 |
| 64 | 81 | 78 | 73 | 79 | 49 | 47 | 45 | 48 |
| 65 | 82-83 | 79 | 74 | 80 | 50 | 48 |  |  |
| 66 | 84 | 80 | 75 | 81 |  |  | 46 | 49 |
| 67 | 85 | 81 | 76 | 82 | 51 | 49 |  | 50 |
| 68 | 86 | 82 | 77 | 83-84 | 52 | 50 | 47 |  |
| 69 | 87 | 83 |  | 85 | 53 |  | 48 | 51 |
| 70 | 88 | 84 | 79 | 86 |  | 51 |  | 52 |
| 71 | 89 | 85 | 80 | 87 | 54 |  | 49 | 53 |
| 72 | 90 | 86 |  | 88 | 55 | 52 | 50 |  |
| 73 | 91-92 | 87 | 82 | 89 |  |  |  | 54 |
| 74 | 93 | 88 | 83 | 90 | 56 |  |  | 55 |
| 75 | 94 | 89 | 84 | 91 | 57 | 54 | 52 |  |
| 76 | 95 | 90 | 85 | 92 | 58 | 55 |  | 56 |
| 77 | 96 |  | 86 | 93-94 |  |  |  | 57 |
| 78 | 97 | 92 |  | 95 | 59 | 56 |  |  |
| 79 | 98 |  |  | 96 | 60 | 57 |  | 58 |
| 80 | 99 |  | 89 | 97 | 61 |  |  | 59 |
| 81 | 101 | 95 |  | 98 |  | 58 |  | 60 |
| 82 | 102 | 96 |  | 99 |  |  |  |  |
| 83 | 103 | 97 |  | 100 |  |  |  | 61 |
| 84 |  | 98 | 93 | 101 |  | 60 |  |  |
| 85 | 105 |  |  | 102 |  | 61 |  |  |
| 86 |  | 100 |  | 103 |  |  |  |  |
| 87 | 107 |  |  | 105 |  |  |  |  |
| 88 |  |  |  |  |  |  | 60 |  |
| 89 |  |  |  | 107 |  |  |  |  |

**The percentile rank norms of the Social Health Scale for the Elderly**

| Percentile rank (%) | Raw score | | | | | | | |
| --- | --- | --- | --- | --- | --- | --- | --- | --- |
|  | Long form | | | | Short form | | | |
|  | 60-69 years | 70-79 years | 80+ years | Total | 60-69 years | 70-79 years | 80+ years | Total |
| <5 | <49 | <48 | <44 | <48 | <28 | <28 | <26 | <28 |
| 5-9 | 49-51 | 48-50 | 44-46 | 48-50 | 28-29 | 28-30 | 26-27 | 28-29 |
| 10-14 | 52-54 | 51-53 | 47-48 | 51-52 | 30-32 | 31 | 28-29 | 30-31 |
| 15-19 | 55-56 | 54-55 | 49-50 | 53-54 | 33 | 32-33 | 30 | 32 |
| 20-24 | 57-58 | 56 | 51 | 55-56 | 34 |  | 31 | 33 |
| 25-29 | 59 | 57-58 | 52-53 | 57-58 | 35 | 34 | 32 | 34 |
| 30-34 | 60-61 | 59 | 54-55 | 59 | 36 | 35 | 33 | 35 |
| 35-39 | 62 | 60-61 | 56 | 60-61 | 37 | 36 | 34 | 36 |
| 40-44 | 63-64 | 62 | 57 | 62 | 38 | 37 | 35 | 37 |
| 45-49 | 65 |  | 58 | 63 | 39 | 38 |  | 38 |
| 50-54 | 66 | 63-64 | 59 | 64 |  | 39 | 36 | 39 |
| 55-59 | 67 | 65 | 60-61 | 65 | 40 |  | 37 | 40 |
| 60-64 | 68-69 | 66 | 62 | 66-67 | 41 | 40 | 38 |  |
| 65-69 | 70 | 67 | 63 | 68 | 42 | 41 | 39 | 41-42 |
| 70-74 | 71-72 | 68-69 | 64 | 69-70 | 43 | 42 | 40 |  |
| 75-79 | 73-74 | 70 | 65-66 | 71-72 | 44-45 | 43 | 41 | 43-44 |
| 80-84 | 75-76 | 71-73 | 67-68 | 73-74 | 46-47 | 44 | 42 | 45 |
| 85-89 | 77-80 | 74-77 | 69-71 | 75-78 | 48 | 45-46 | 43-44 | 46-47 |
| 90-94 | 81-87 | 78-82 | 72-76 | 79-84 | 49-51 | 47-49 | 45-46 | 48-50 |
| 95- | 88- | 83- | 77- | 85- | 52- | 50- | 47- | 51- |
